# An online soft sensor method for biochemical reaction process based on JS-ISSA-XGBoost

**DOI:** 10.1186/s12896-023-00816-3

**Published:** 2023-11-08

**Authors:** Ligang Zhang, Bo Wang, Yao Shen, Yongxin Nie

**Affiliations:** School of Electrical and Information Engineering, JiangSu University, ZhenJiang, 212013 JiangSu China

**Keywords:** Online soft sensor, Just-in-time learning strategy, eXtreme gradient boosting method, *Pichia pastoris*

## Abstract

**Background:**

A method combining offline techniques and the just-in-time learning strategy (JITL) is proposed, because the biochemical reaction process often encounters changing features and parameters over time.

**Methods:**

Firstly, multiple sub-databases in the fermentation process are constructed offline by an improved fuzzy C-means algorithm and the sample data are adaptively pruned by a similarity query threshold. Secondly, an improved eXtreme Gradient Boosting (XGBoost) method is used on the online modeling stage to build soft sensor models, and the multi-similarity-driven just-in-time learning strategy is used to increase the diversity of the model. Finally, to improve the generalization of the whole algorithm, the output of the base learner is fused by an improved Stacking integration model and then the predictive output is performed.

**Results:**

Applying the constructed soft sensor model to the problem of predicting cell concentration and product concentration in *Pichia pastoris* fermentation process. The experimental results show that the root mean square error of the cell concentration is 0.0260, the coefficient of determination is 0.9945, the root mean square error of the product concentration is 2.6688, and the coefficient of determination is 0.9970. It shows that the proposed method has the advantages of timely prediction and high prediction accuracy, which validates the effectiveness and practicality of the method.

**Conclusion:**

The JS-ISSA-XGBoost is an extensive and excellent soft measurement model that meets the practical needs for real-time monitoring of parameters and prediction of control in biochemical reactions.

## Background

With the development of science and technology, the monitoring and control of biochemical reaction parameters are more and more demanding. However, the biochemical reaction process is a multivariate, time-varying, uncertain, and strongly coupled nonlinear system [1–3]. Due to the actual process technology and production cost, key biochemical parameters cannot be directly measured online and can only be roughly estimated by offline sampling and analysis. This process not only causes a lag in the acquisition of information, which can affect the operator's ability to make correct judgments and decisions about the real-time response status, but it also limits the implementation of optimal control strategies. Therefore, there is an urgent need to find a method to achieve optimal estimation and prediction of key biochemical parameters in the biochemical reaction process.

Soft sensor method is an effective way to solve the problem of difficult online measurement of key biochemical parameters in reaction processes, e.g., Yu proposed a soft sensor modeling framework combining Bayesian inference strategies and support vector machines (SVR) and applied it to predict the production of biological processes [4]. Mei et al. proposed a method based on the combination of the Gaussian regression model (GPR) and principal component analysis (PCA) to estimate the biomass of the erythromycin fermentation process [5].Wang et al. proposed a recurrent wavelet neural network (RWNN) and Gaussian process regression (GPR) based method to develop a soft sensor model for online measurement of fermentation broth parameters and total sugar content for online prediction of whether aureomycin fermentation broth is contaminated with non-target bacteria [6]. However, most of the soft sensor models developed in the above literature use offline modeling methods. Although soft sensor models can greatly improve the prediction in real-time in practical applications, the characteristics of the actual biochemical reaction process and the relevant parameters change over time, and there is a risk that the offline modeling methods will fail in different reaction environments. Moreover, offline models need to be retrained at regular intervals, which is costly in terms of time and not conducive to long-term use in actual biochemical reaction production. The Just-in-Time Learning(JITL) strategy has attracted much attention from the academic community to address the problems of offline modeling methods [7]. For example, Ren et al. used locally weighted partial least squares (LWPLS) with a Just-in-Time Learning strategy for industrial soft sensor modeling and showed that this method has a higher prediction accuracy than other methods [8]. Yuan et al. proposed an adaptive soft sensor modeling method based on the moving window (MW) and JITL techniques [9]. The results show that the proposed soft sensor of non-linear time-varying processes has a high prediction accuracy. Although the above online local modeling methods take advantage of the high accuracy of online modeling predictions from JITL, they do not consider the real-time nature of the speed of online modeling predictions. When the training samples are large, the real-time performance of the model will be seriously affected, making it difficult to be widely used in biochemical reaction process.

Considering the above problems, this paper proposes a soft sensor modeling method combining offline techniques and Just-in-Time Learning (JITL) strategy. First, multiple query domains are constructed offline by an improved fuzzy C-mean algorithm, and query thresholds adaptively prune the sample data. Secondly, an improved eXtreme Gradient Boosting (XGBoost) method is used to build soft sensor models in the online modeling stage, and the multi-similarity-driven JITL scheme is used to increase the diversity of the models. Finally, to improve the generalization of the whole algorithm, the output of the base learner is fused by an improved Stacking integration model and then the predictive output is performed. Applying the constructed soft sensor model to the problem of predicting cell concentration and product concentration in *Pichia pastoris* fermentation process. The experimental results show that the proposed method has the advantages of timely prediction and high prediction accuracy, which validate the effectiveness and practicality of the method.

## Methods

### Local query domain creation method in offline phase

#### Improved FUZZY C-mean (IFCM) algorithm

Traditional soft sensor modeling methods based on just-in-time learning usually involve a cumbersome process of selecting similar sample points across the entire sample dataset. When the historical data set is too large, it will lead to long search times for the algorithm, making it impossible for the soft sensor model to predict the output on time.

In particular, the distribution of sample data in the time-varying continuous non-linear process of biochemical reactions is not concentrated, and the predictive performance of the model is limited by using all relevant samples to build a single overall model. Therefore, this paper uses the Improved Fuzzy C-mean (IFCM) algorithm to divide the queried domain to address the above issues.

Firstly, considering that the traditional fuzzy C-mean algorithm (FCM) [10–13] suffers from sensitivity to the initial centroid of clustering and a high number of iterations, this paper proposes an enhanced algorithm to improve the FCM (IFCM). The initial values of the FCM algorithm are usually set artificially, and the model is prone to fall into local optimality. This paper determines the number of classifications by the "elbow method" [14] to avoid human intervention. In addition, the distance between all data sample points and the origin is calculated using the Mahalanobis distance, as shown in Eq. (1).

1 $${D}_{M}\left(x,y\right)=\sqrt{{\left(x-y\right)}^{T}{\sum }^{-1}\left(x-y\right)}$$

where: $\sum$ denotes the covariance matrix of the covariance matrix of the multidimensional random variables of $x$ and $y$.

The core metric of the elbow method is the sum of squared errors (SSE), which is used to represent the clustering error. As the number of clusters $k$ increases, the sample division will become finer, the degree of aggregation of each cluster will gradually increase, and the SSE will naturally become progressively smaller. Moreover, when $k$ is less than the actual number of clusters, the decrease in SSE will be significant because an increase in $k$ will substantially increase the degree of aggregation of each cluster. However, when $k$ reaches the actual number of clusters, the return on the degree of aggregation obtained by increasing $k$ will decrease rapidly, so the decline in SSE will decrease sharply and then level off as the value of $k$ continues to increase. This means that the graph of the relationship between SSE and $k$ is the shape of an elbow, and the value of *k* corresponding to this elbow is the actual number of clusters of the data.

Secondly, the sample points are ranked according to the Mahalanobis distance, and the clustering subsets are divided equally. In each clustering subset, the middle sample point is selected as the initial clustering center of the FCM algorithm, and the local query domain is constructed.

**Fig. 1:**
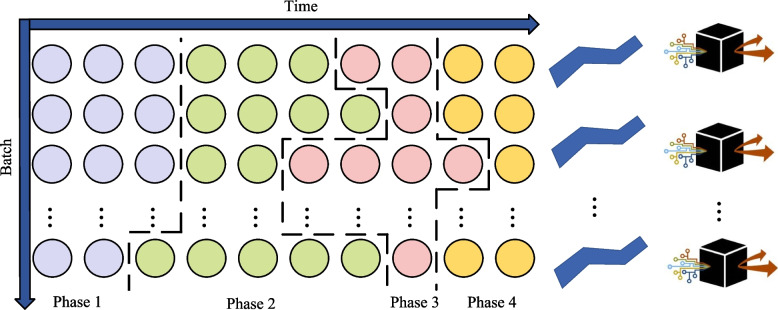
Partitioning of local query domains based on the IFCM algorithm

Finally, the FCM algorithm's affiliation matrix divides the historical sample dataset into a reasonable number of sub-databases, as shown in Fig. 1. Each of these sub-databases is a local query domain for JITL. By creating a local query domain, the search range of the algorithm is made smaller, and the objective function of the FCM is shown in Eq. (2).

2 $${J}_{m}=\sum_{i=1}^{n}\sum_{j=1}^{c}{u}_{ij}^{m}{\Vert {x}_{i}-{v}_{j}\Vert }^{2},2\le m<\infty$$

where: $\Vert {x}_{i}-{v}_{j}\Vert$ is the Euclidean distance from the sample point $x_i$ to the centroid $v_j$, $u_{ij}$ is the affiliation function. $m\left(m>1\right)$ is the fuzzy index, and generally taken as *m* = 2. *n* denotes the number of populations and $c$ denotes the number of samples.

As the data samples of actual biochemical reaction process present a high-dimensional non-linear distribution, the Euclidean distance in the traditional FCM algorithm has some advantages for spherical structure clustering. However, some computational disadvantages exist in solving a high-dimensional data problem like to the biochemical reaction process. Therefore, the IFCM algorithm can cluster the sample data more accurately and consistently than the direct use of the FCM algorithm.

#### Adaptive pruning database

**Fig. 2:**
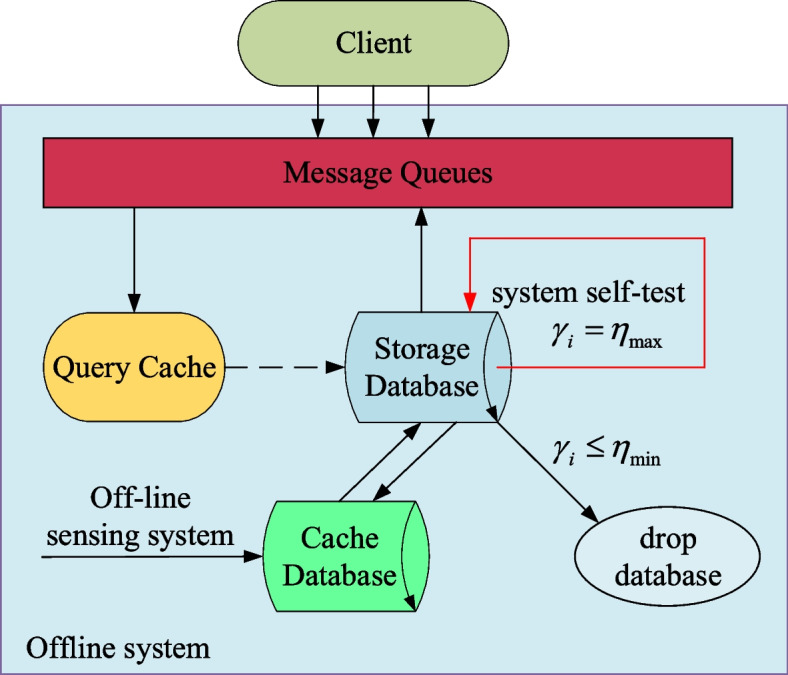
The offline system dynamically updates the database

In the actual biological biochemical reaction process, the accumulation of sample data in the sub-database over time can seriously affect the response rate of the JITL model. Aiming at this problem, this paper proposes an adaptive pruning data mechanism to update the sample data in the database automatically to address such problems. The specific mechanism is as follows:

A similarity query label $\gamma_i$ is created in each sub-database and a minimum threshold ${\eta }_{\mathrm{min}}$ and a maximum threshold ${\eta }_{\mathrm{max}}$ for the number of similarity queries is set, which increases when the output samples ${x}_{i}$ in the sub-database are involved in immediate learning. When ${\gamma }_{i} = {\eta }_{\mathrm{max}}$ exists in the sub-database, the current database automatically deletes all samples of ${\gamma }_{i} \le {\eta }_{\mathrm{min}}$ and re-updates all previous predicted data results and corresponding auxiliary variables to the current database, and then initializes the value of ${\gamma }_{i}$, as shown in Fig. 2.

The adaptive data pruning mechanism prunes the data in the database well and dynamically maintains the quantities in the biochemical reaction process sub-database so that the data in the sub-database can meet the requirements of the JITL strategy.

#### Dynamic filtering of query domain auxiliary variables

Considering many auxiliary variables measured online during the biochemical reaction process, some of the auxiliary variables do not correlate well with the dominant variables, and too many input variables increase the complexity of the model and reduce its response speed. In addition, as the process characteristics and various parameters change in different stages of biochemical reaction process, the auxiliary variables representing the dynamic characteristics of the biochemical reaction process will change accordingly. This paper uses the K-nearest neighbor mutual information estimation (K-MI) method for dynamic real-time screening of auxiliary variables to improve the predictive performance of the soft sensor model.

Mutual information was first proposed by Shannon [15]. Intuitively, mutual information can measure the interrelationship between two random variables. However, the probability distribution of each variable is unknown in the actual soft sensor implementation, making the calculation of mutual information difficult. However, the probability distribution of each variable is unknown in the actual soft sensor implementation, making the calculation of mutual information difficult. Therefore, the mutual information between variables can be estimated directly using the K-nearest neighbor estimation of mutual information (K-MI) method [16–18]. The estimate of the mutual information $I\left(x,y\right)$ is:

3 $$I\left(x,y\right)=\Psi \left(k\right)-1/k-\langle \Psi \left({n}_{x}\right)+\Psi \left({n}_{y}\right)\rangle +\Psi (N)$$

where: $\Psi \left( k \right)$ is the digamma function, $\Psi \left( x \right) = {\Gamma^{ - 1}}\left( x \right)d\Gamma \left( x \right)/dx$; $k$ is generally considered to be 2 to 6, and ${k}$ is set to be 4 in this circle. $N$ is the number of samples. $\langle\cdots\rangle$ means that the values of the digamma function are averaged over all variables, i.e., $\langle \cdots \rangle ={N}^{-1}\sum\nolimits_{i=1}^{N}E\left[\cdots (i)\right]$.

K-MI algorithm is used to filter auxiliary variables, which not only reduces the model's complexity and improves the model's response time, but also contributes to the predictive performance of the model.

### JITL strategy in online modeling phase

#### JITL principles

**Fig. 3:**
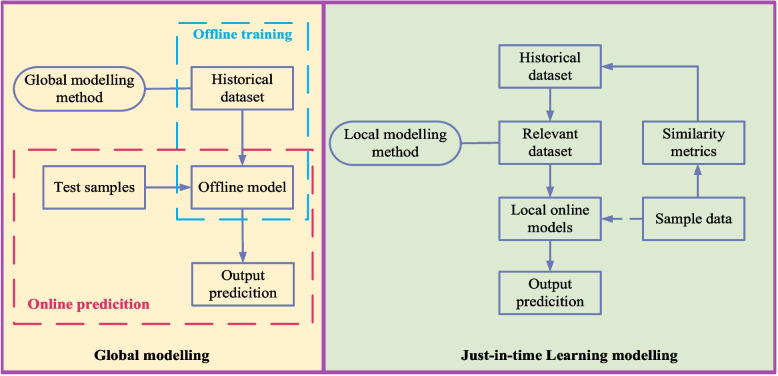
Comparison between traditional modeling methods and JITL

JITL is an online local modeling method. which builds a historical database by collecting a large sample of data offline. When a prediction sample arrives, the prediction model first looks for samples similar it in the historical database, then uses these similar data to build a local model, and finally predicts the output. As soon as the prediction results are output, the model will be immediately abandoned while waiting for the following measurement sample to arrive. The JITL strategy is more suitable for the biochemical reaction process than using a traditional offline global model. The comparison between traditional modeling methods and JITL modeling framework is shown in Fig. 3.

#### JITL method based on multiple similarity metrics

In JITL, A single similarity metric cannot accurately portray the relationship between input and output, resulting in poor model generalization performance. Several weighting functions are available for assessing similarities, such as truncation and Gaussian functions. However, it is pointed out that the selection of weight functions may not influence the modeling performance as the selection of similarity [19]. Hence, the definition of similarity plays a significant role in the success of the JITL modeling framework. Based on this, this paper uses multiple similarity metrics to assess the similarity between samples, allowing for increased model diversity and enhancing model robustness performance. This paper uses Euclidean Distance (ED), Covariance Weighted Distance (CWD), and similarity metrics based on distance and angle to select suitable sample sets.

1. ED Similarity. This metric is defined based on the distance of the data from two points in Euclidean space.

4 $${\omega }_{i} = {e}^{\left(-{d}_{i}^{2}/{\varphi }_{1}{\sigma }_{d}\right)}$$

5 $${d}_{i}=\sqrt{{\left({x}_{i}-{x}_{q}\right)}^{T}\left({x}_{i}-{x}_{q}\right)}$$

where: $d_i$ is the Euclidean distance between the query sample and the historical sample in Eq. (4). in Eq. (5), $\sigma_d$ is the standard deviation of the distance vector ${d}_{i}$ and ${\varphi }_{1}$ is the local adjustment parameter.

(2) CWD Similarity. This metric considers the relationship between input variables and between input and output variables.

6 $${d}_{i}=\sqrt{{\left({x}_{i}-{x}_{q}\right)}^{T}H\left({x}_{i}-{x}_{q}\right)}$$

7 $$H={\left({X}^{T}y\right)}^{T}\left({X}^{T}y\right)/{\Vert {X}^{T}y\Vert }^{2}$$

where:$H$ is the weighting matrix,$X$ and $y$ are the input and output matrices, respectively.

(3) A similarity metric based on distance and angle. The metric uses the angle between two vectors in the space of a sample to measure the degree of similarity between samples.

8 $$\mathrm{cos}\left({\theta }_{i}\right)=\langle {x}_{i},{x}_{q}\rangle / \left({\Vert {x}_{i}\Vert }_{2}{\Vert {x}_{q}\Vert }_{2}\right)$$

9 $${\omega }_{i} = \lambda \sqrt{{e}^{\left(-{d}_{i}^{2}/{\varphi }_{2}{\sigma }_{d}\right)}}+ \left(1-\lambda \right)\mathrm{ cos}\left({\theta }_{i}\right),\mathrm{ cos}\left({\theta }_{i}\right)\ge 0$$

where: ${d}_{2,i}$ and $\mathrm{cos}\left({\theta }_{xi}\right)$ denote the distance and angular similarity between the query and historical samples, respectively.

In this paper, three local models are constructed using three similarity metrics to filter the queried domain and generate diverse local state identification results.

#### Local XGBoost model construction

**Fig. 4:**
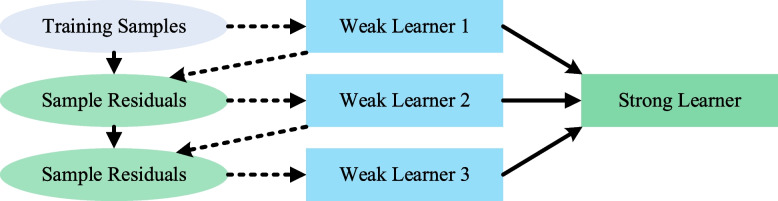
Learning method of XGBoost model

In the online modeling stage, the XGBoost algorithm is chosen as the base learner for soft sensors, considering the stability and rapidty of XGBoost. The XGBoost algorithm is implemented in a gradient boosting framework, where base learners are built during boosting, with each base learner learning from the previous base learner and updating the residuals. A strong learner is eventually formed by analyzing the base learners' learning residuals and updating the sample weights during each iteration, as shown in Fig. 4.

First define a decision tree whose output function is shown in Eq. (10).

10 $$f\left(x\right)={\omega}_{q}\left(x\right), \omega \epsilon {R}^{T} ,q:{R}^{d}\rightarrow\left\{1, 2,\dots ,T\right\}$$

where: $x$ is the input vector, $q$ is the structure of the tree, $\omega$ is the corresponding leaf fraction, $T$ is the number of nodes in the tree with leaves, and $d$ is the dimensionality of the data features. Then, assuming that the fraction of leaf nodes of sample *i* in the $jth$ decision tree is $\omega_{ij}$, the output function of this sample after *t* decision tree iterations is given by Eq. (11). The objective function of the XGBoost algorithm is shown in Eq. (12).

11 $${\widehat{y}}_{l}^{\left(t\right)}=\sum_{j=1}^{t}{f}_{j}\left({x}_{i}\right)=\sum_{j=1}^{t}{\omega }_{ij}$$

12 $${Obj}^{\left(t\right)}=\sum_{j=1}^{N}L\left({y}_{i},{\widehat{y}}_{l}^{\left(t\right)}\right) +\sum_{j=1}^{t}\Omega \left({f}_{j}\right)$$

where: $$\sum {_{i = 1}^NL\left( {{y_i},\overset{\lower0.5em\hbox{$\smash{\scriptscriptstyle\frown}$}}{y}_l^{\left( t \right)}} \right)}$$ is the loss function, which represents the sum of the error values between the true value $$y_i$$ and the predicted value $${\widehat{y}}_{l}^{\left(t\right)}$$.

Assuming that the XGBoost algorithm does not constrain the number of nodes, the tree's structure splits maximally, in which case the XGBoost model will be overfitted. Therefore, a regular term $\Omega \left({f}_{j}\right)$ is added to the objective function to prevent over-fitting. A penalty term ζ is introduced into the objective function of a single decision tree, as shown in Eq. (13).

13 $$\Omega \left({f}_{t}\right)=\gamma T +\frac{1}{2} \lambda \sum_{j=1}^{T}{\omega }_{j}^{2}$$

where: $T$ is the number of leaf nodes. $\omega_j$ is the fraction of the $jth$ leaf node. $\gamma$ and $\lambda$ are hyperparameters to control the generalization error and prevent overfitting.

It should be noted that the XGBoost algorithm uses a second-order Taylor expansion for the loss function, which not only improves the accuracy of the model but also allows the gradient to converge faster, just as Newton's method converges faster than SGD. After simplification and $t$ iterations, the objective function is as follows:

14 $${obj}^{(t)} =\gamma T + \sum_{j=1}^{T}\left({\omega }_{j}{G}_{j}+ \frac{1}{2} {\omega }_{j}^{2} \left(\lambda +{H}_{j}\right)\right)$$

where: $G_j$ is $\sum {_{i \in {I_j}}{g_i}}$, ${H_j}$ is $\sum {_{i \in {I_j}}{h_i}}$.

 To find the optimal solution of the objective function, the minimum value of $Obj^{(t)}$ is required, i.e., the minimum value of ${\omega }_{j}$ is found in ${\omega }_{j}^{*}$. The optimal solution of the objective function is equivalent to Eq. (15), and the final optimal solution of the objective function is obtained as shown in Eq. (16).

15 $${\omega }_{j}^{*}=\mathrm{arg\,min}\left({\omega }_{j}{G}_{j} +\frac{1}{2}{\omega }_{j}^{2}\left(\lambda +{H}_{j}\right)\right)$$

16 $${{Obj}^{\left(t\right)}}^{*}=\gamma T-\frac{1}{2}\sum_{j=1}^{T}\frac{{G}_{j}^{2}}{\lambda +{H}_{j}}$$

In addition, XGBoost models are engineered to support parallelized model training, and the problem of not being able to load all the feature values into local memory for distributed datasets can be solved by XGBoost models using an approximate histogram algorithm. At the same time, the XGBoost algorithm's cache-aware access technology and Block out-of-core compute optimization technology can efficiently increase the system's resource usage. These engineering optimizations specific to XGBoost models can all significantly improve the speed of XGBoost modeling. The use of the XGBoost model as a base learner is very suitable for the JITL strategy compared to other models.

#### Improved sparrow algorithm

In the modeling process, the accuracy and robustness of the free-growing XGBoost model are easily affected by the parameters, and allowing the XGBoost model to grow freely will result in an over-fitting model. In addition, although the free-growing XGBoost model will improve the model prediction accuracy, it will significantly reduce the model's computational efficiency, increase the system's lag, and is unsuitable for online modeling strategy. Therefore, parameters such as the learning rate, the maximum number of iterations, and the maximum depth of the tree of XGBoost need to be optimized as a way of balancing all aspects of the performance of the XGBoost model, i.e., to improve the convergence speed of the model without losing prediction accuracy. The Sparrow Search Algorithm (SSA) has been widely used among the various algorithms for optimizing parameters. It is a new intelligent optimization algorithm that mainly simulates the foraging and predation prevention process of a sparrow flock [20], and consists of a sparrow flock foraging model with a discoverer, a follower, and an early warning. The specific search process is as follows:

1. The mathematical expression for the iterative update of the discoverer position is shown in Eq. (17).
  17 $${x}_{i,d}^{t+1}=\begin{array}{c}\left\{\frac{-i}{\alpha \cdot {g}_{max}}\right.,r<\beta \\ {x}_{i,d}^{t+1}+q \cdot l, r\le \beta \end{array}$$
2. The mathematical expression for iterative follower position update is shown in Eq. (18).
  18 $${x}_{i,d}^{t+1}=\left\{\begin{array}{c}q\cdot \mathrm{exp}\left(\frac{{x}_{worst}^{t}-{x}_{i,d }^{t}}{{t}^{2}}\right),i\geq \frac{n}{2}\\ {x}_{p}^{t+1}+\left|{x}_{i,d}^{t}-{x}_{p}^{t+1}\right|\cdot {a}^{+} \cdot l, i\le \frac{n}{2}\end{array}\right.$$
3. The mathematical expression for the anti-predatory behavior of an early warning when it becomes aware of danger is shown in Eq. (19).
  19 $${x}_{i,d}^{t+1}=\left\{\begin{array}{c}{x}_{best}^{t+1} + \rho \cdot \left|{x}_{i,d}^{t}-{x}_{best}^{t}\right|,{f}_{i} > {f}_{g}\\ {x}_{i,d}^{t}+k\cdot \left(\frac{\left|{x}_{i,d}^{t}-{x}_{best}^{t}\right|}{\left({f}_{i}-{f}_{\omega }\right)+\varepsilon }\right), {f}_{i} ={f}_{g}\end{array}\right.$$

It is worth noting that sparrow populations require extensive optimization searching in the early iterations. At the same time, diversity decreases in late iterations, leading to premature algorithm convergence and a tendency to fall into local extremes. To address this problem, this paper proposes a hybrid variational optimization strategy (ISSA), i.e., using the standard Cauchy distribution function and standard Gaussian distribution function to enhance the diversity of the sparrow population so that the joiners have a more vital ability to jump out of the optimal local solution.

The hybrid variation strategy introduces dynamic variation parameters ${\lambda }_{1}$, ${\lambda }_{2}$ according to the number of iterations.

20 $${x}_{i,d}^{{t+1}{\prime}}= {x}_{best}^{t+1}\left[1+{\lambda }_{1} Cauchy\left(\mathrm{0,1}\right) +{\lambda }_{2} Gauss\left(\mathrm{0,1}\right)\right]$$

21 $${\lambda }_{1} =1-\frac{{t}^{2}}{{T}^{2}}$$

22 $${\lambda }_{2} =\frac{{t}^{2}}{{T}^{2}}$$

where: $t$ is the current number of iterations; $T$ is the maximum number of iterations; and the standard Gaussian distribution function and standard Cauchy distribution function are shown below:

23 $$f\left(x\right)=\frac{1}{\sqrt{2\pi }}\mathrm{exp}\left(-\frac{{x}^{2}}{2}\right) -\infty <x <+\infty$$

24 $$f\left(x\right)=\frac{1}{\pi \left(1+{x}^{2}\right)} -\infty <x <+\infty$$

The hybrid variation strategy is to generate a new location after each iteration based on the joiner location in the current iteration and to compare the fitness values of the two locations. During the iterative process, parameter ${\lambda }_{1}$ is gradually reduced, and parameter ${\lambda }_{2}$ is gradually increased, thus enhancing the ability of the algorithm to jump out of local extremes and global search. This paper uses the ISSA algorithm to optimize the XGBoost model, resulting in superior robustness and predictive power.

**Fig. 5:**
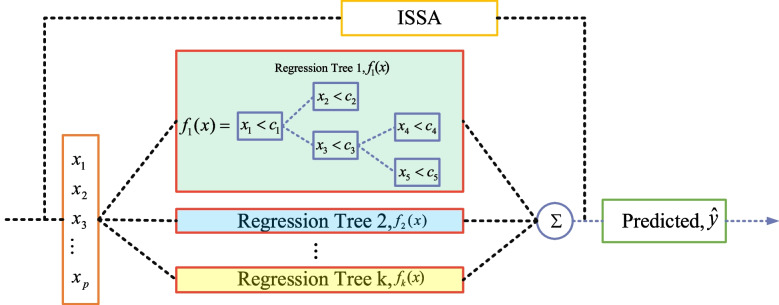
Structure of the ISSA algorithm for optimizing the XGBoost model

The structure of the ISSA algorithm for optimizing the XGBoost model is shown in Fig. 5.

#### Model stacking strategy based on multilayer perceptron

**Fig. 6:**
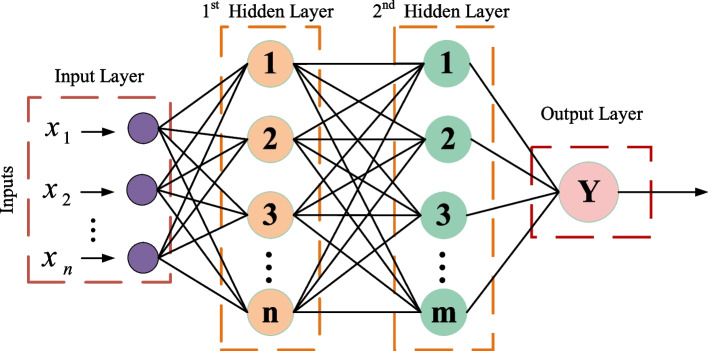
Structure diagram of multilayer perceptron

Considering the multiple XGBoost primary learning models established in JITL modeling of similar metrics, it is necessary to further integrate multiple XGBoost models. Currently, most multi-model fusions use the weighting approach in the integration strategy to determine the models' weights by cross-validation. However, cross-validation does not guarantee the best model selection in terms of the actual generalization performance of the test set [21]. In order to enhance the generalization performance of the whole soft sensor model, this paper uses model stacking strategy to improve the prediction performance of the soft sensor model. At the same time, to prevent the model from overfitting, using a weakly fitted multilayer perceptron (MLP) as the second layer of the meta-learner, the structure of the MLP is shown in Fig. 6.

As shown in Fig. 6, a multilayer perceptron model with a forward structure is constructed, and the complexity of the model is balanced by adjusting the number of hidden layers and the number of neurons. When the model is overfitting, the model's generalization ability can be increased by reducing the number of hidden layers and the number of neurons in the MLP model. Conversely, when the model appears to be under-fitted, the model complexity can be increased by increasing the number of hidden layers and neurons in the MLP model.

**Fig. 7:**
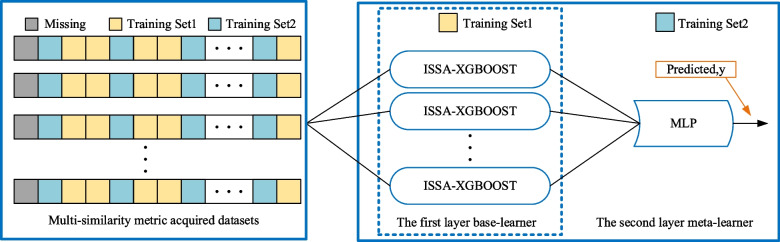
The construction of the Stacking model and the training method of the model

In addition, the training set for the secondary learners in most Stacking model research strategies will also be obtained using k-fold cross-validation. However, for the free-growing XGBoost model, the k-fold cross-validation approach does not substantially improve the generalization of the metamodel, and there is a risk of data leakage. It takes several experimental simulations to find the exact number of k-folds, which greatly wastes time for model construction. Therefore, this paper adopts a new strategy to optimize the Stacking model, replacing the K-fold cross-validation scheme by pre-separating the data set. Firstly, the data set is obtained through multiple similarity measures, and the similar data sets of each model are arranged according to the similarity; Then, a portion of the data set is extracted using uniform sampling, which allows a greater degree of information about the characteristics of the data to be obtained; Finally, the separated data set is used as the training set of the meta-learner, as shown in Fig. 7. This paper uses an optimized solution that is more adapted to the JITL strategy than the original cross-validation (CV) solution in Stacking, which not only dramatically prevents the reuse of data and reduces the risk of information leakage but also allows the system to be more responsive.

### Modeling process

**Fig. 8:**
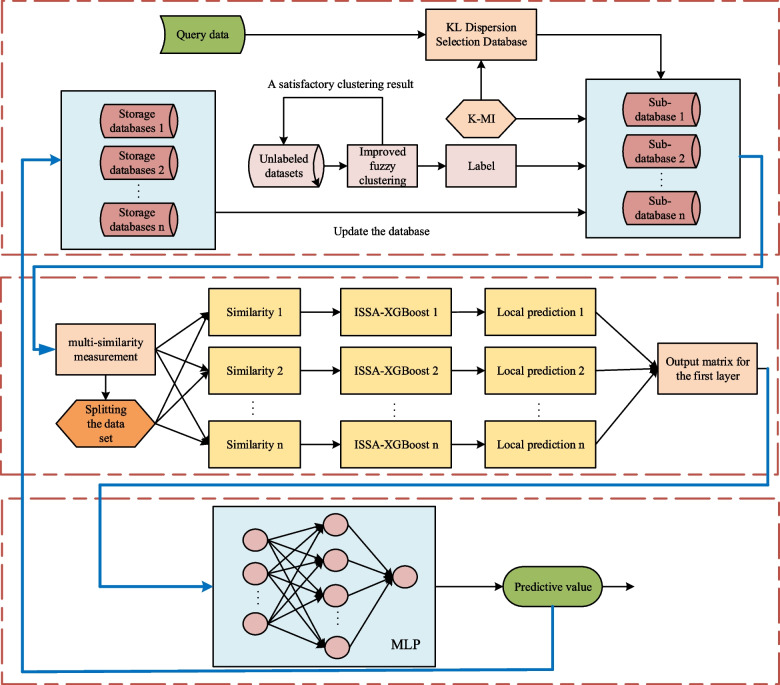
Overall structure diagram of modeling algorithm

The flow of the modeling method proposed in this paper is shown in Fig. 8.

To better illustrate the process of online soft sensor modeling in this paper, the modeling process is described as follows:

- Step 1: In the offline stage, multiple query domains are divided using the IFCM algorithm, and the main auxiliary variables in each query domain are determined separately using the K-MI algorithm.
- Step 2: In the online prediction stage, the KL scatter is used to determine the sub-database in which the queried domain is located when the query data arrives.
- Step 3: The multi-similarity measure is used to extract the data in the queried domain, and the extracted data is sorted and segmented. Finally, the remaining data after segmentation are fed into the ISSA-XGBoost local algorithm for model training, respectively.
- Step 4: The separated dataset is also fed into the ISSA-XGBoost model, and the matrix results predicted by the first layer of the model are then fed into the MLP algorithm for MLP model training.
- Step 5: Send the query data to the JS-ISSA-XGBoost model for prediction output, and store the output results in the storage database to wait for the sub-database update.
- Step 6: When the query data obtains the current prediction result through the JS-ISSA-XGBoost model, the query data and the JS-ISSA-XGBoost model will be released, and the system waits for the arrival of the following query data.

## Results

### Simulation example

**Fig. 9:**
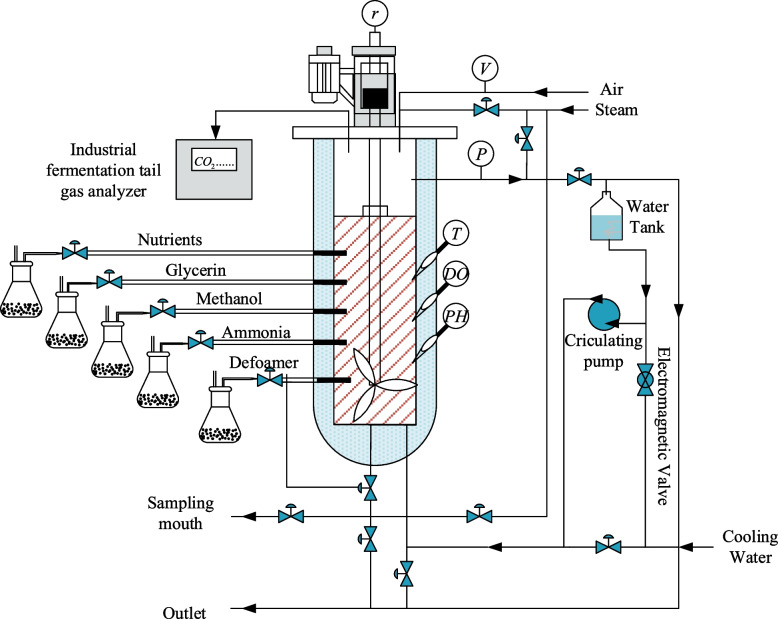
Structure of Pichia pastoris fermentation process

In order to validate the effectiveness of the proposed online soft sensor modeling method, this paper simulates the data from *Pichia pastoris* fermentation process. *Pichia pastoris* fermentation process is a highly coupled multi-input and multi-output system, which has the characteristics of high nonlinearity, time-varying and hysteresis. Due to the difficulty of measuring some key biochemical parameters online, the *Pichia pastoris* fermentation process cannot be effectively controlled and optimized [22, 23]. Therefore, it is of great significance to establish an effective soft sensor model for *Pichia pastoris* fermentation process. Taking *Pichia pastoris* fermentation as the object, the *Pichia pastoris* GS115, Mut^S^His + was selected as the strain in this experiment. The fermentation process was completed on the pilot platform provided by Yangzhong Jiaocheng Biotechnology Research Co., Ltd, and the adopted fermentation tank was the A103-500L model. Figure 9 shows the structure of *Pichia pastoris* fermentation process.

The fermentation period is approximately 90 h, and the sampling period of auxiliary variables is 15 min. This paper builds a database by uploading the collected data to a computer through a distributed control system. In addition, the cell concentration $X$ and product concentration $P$ were measured by a spectrophotometer, and the sampling period was determined according to the situation. During the exponential growth phase, offline sampling and measurement were performed every 1 h, and sampling was performed every two hours during other periods. The cell concentration was measured by the dry cell weight method: 10 ml of the fermentation broth was centrifuged at 10000r/min for 10 min in a TGL20M high-speed centrifuge. After removing the supernatant, the pellet was washed twice with deionized water, dried at 80 °C to constant weight, and then weighed.

The environmental variables measured by the sensing instruments were taken much more frequently than those sampled offline during building the initial database. In order to align critical parameters such as cell concentration with the sampling time of the environmental variables, this paper uses interpolation to supplement them. The fermentation data of 10 batches of *Pichia pastoris* were collected through experiments. 3600 samples were obtained by interpolation method, 90% of which were used as the training set and 10% as the test set.

### Query domain split creation

**Fig. 10:**
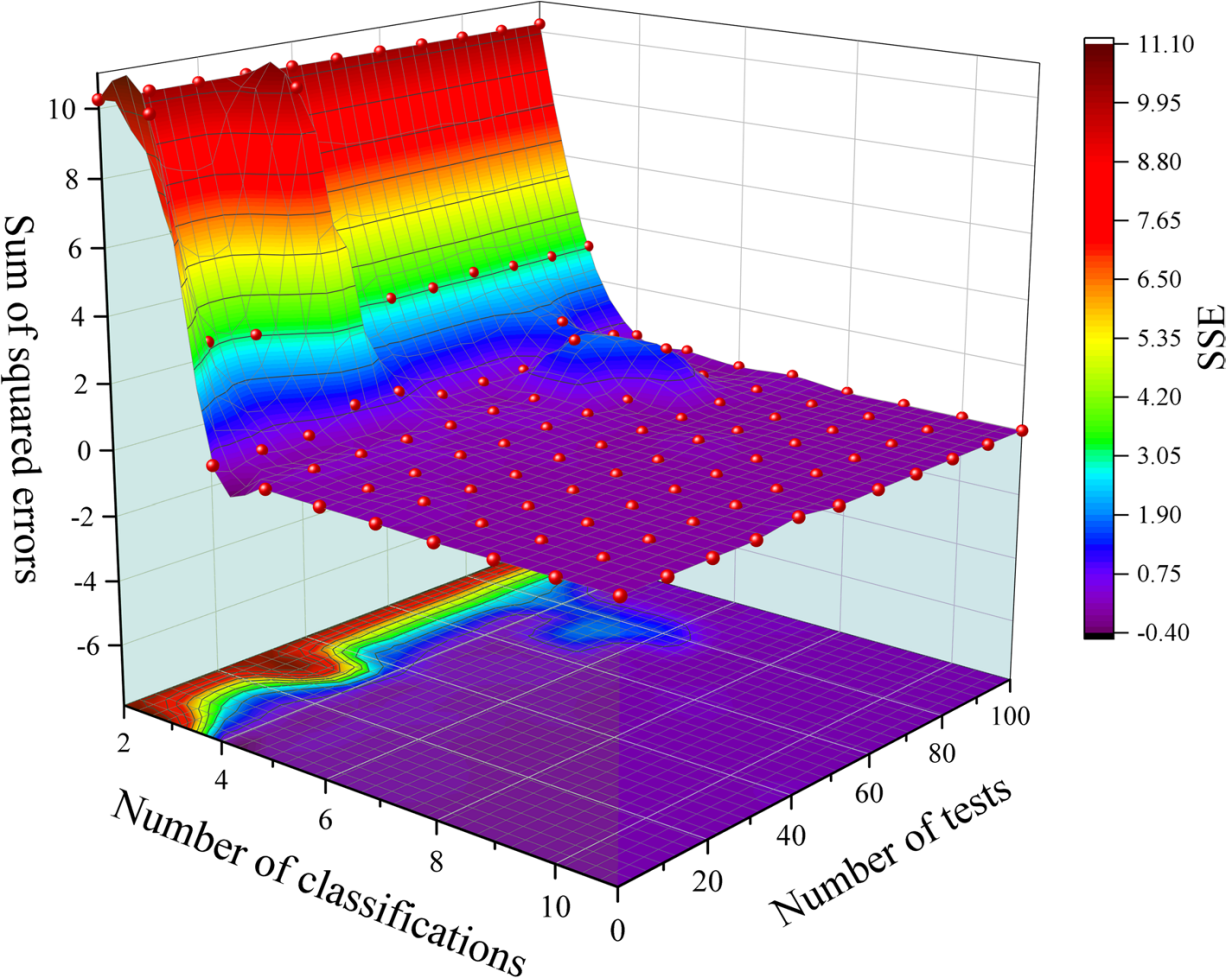
SSE values for different numbers of clusters

The "elbow method" was used to determine the number of divisions. After several tests, when $k>4$, the downward trend tends to level off, so the optimal number of clustering centers is 4, i.e., the offline state of the queried domain to be divided into four sub-databases, as shown in Fig. 10.

### Local variable selection

By analyzing the mechanism of *Pichia pastoris* fermentation process, it can be known that the cell concentration and the product concentration can reflect the internal state of the fermentation to the greatest extent, so these two variables are selected as dominant variables that need to be predicted. The environmental variables that can be measured directly by the instrument during fermentation are Dissolved oxygen concentration $\left(DO\right)$, Exhaust ${CO}_{2}$ concentration $\left({\eta }_{{CO}_{2}}\right)$, $pH$ of fermentation broth $\left(pH\right)$, Flow acceleration rate of inorganic salts ${f}_{b}$, Pressure in the fermenter $\left(P\right)$, Flow of air $\left(l\right)$, Fermentation time $\left(t\right)$, Flow rate of condensate ${f}_{w}$, Flow acceleration rate of ammonia ${f}_{a}$, Flow acceleration rate of methanol ${f}_{f}$, Flow acceleration rate of glucose ${f}_{c}$, Fermentation temperature $\left(T\right)$, Motor stirring speed $\left(r\right)$, Flow acceleration rate of glycerine $\left({f}_{e}\right)$, Flow acceleration rate of peptones ${f}_{d}$, Volume of fermentation broth $\left(V\right)$. These measurable variables are used as auxiliary variables, and the mutual information between the auxiliary and dominant variables in different query domains is calculated as shown in Table 1.

**Table 1:** Mutual information between environmental and dominant variables

Environmental variables	Query Domain			
	QD1	QD2	QD3	QD4
Dissolved oxygen concentration $\left(DO\right)$	1.16	1.23	1.11	1.09
Exhaust ${CO}_{2}$ concentration ${\eta }_{{CO}_{2}}$	1.05	1.10	1.13	1.07
$pH$ of fermentation broth $\left(pH\right)$	0.65	0.62	0.61	0.59
Flow acceleration rate of inorganic salts $\left({f}_{b}\right)$	0.73	0.72	0.69	0.71
Pressure in the fermenter $\left(P\right)$	0.12	0.21	0.10	0.32
Flow of air $\left(l\right)$	0.21	0.36	0.35	0.32
Fermentation time $\left(t\right)$	1.39	1.45	1.30	1.35
Flow rate of condensate ${f}_{w}$	0.90	0.96	1.05	0.97
Flow acceleration rate of ammonia $\left({f}_{a}\right)$	0.85	0.79	0.72	0.71
Flow acceleration rate of methanol $\left({f}_{f}\right)$	0.83	1.21	1.04	0.80
Flow acceleration rate of glucose ${f}_{c}$	0.44	0.82	0.77	0.81
Fermentation temperature $\left(T\right)$	0.31	0.35	0.29	0.32
Motor stirring speed $\left(r\right)$	0.19	0.23	0.2	0.17
Flow acceleration rate of glycerine $\left({f}_{e}\right)$	0.46	0.81	0.86	0.73
Flow acceleration rate of peptones $\left({f}_{d}\right)$	0.28	0.35	0.41	0.39
Volume of fermentation broth $\left(V\right)$	0.76	0.71	0.76	0.75

The mutual information in Table 1 of this paper was arranged in order, and the top 6 environmental variables were selected as auxiliary variables for each query domain. Each query domain constructs its soft sensor model, as shown in Eq. (25).

25 $$\left\{\begin{array}{c}\begin{array}{c}{\Phi }_{1} \left(X\right) =f\left(t, DO,{\eta}_{{CO}_{2}},{f}_{w},{f}_{a},{f}_{f}\right)\\ \begin{array}{c}{\Phi }_{2 }\left(X\right) =f\left(t, DO,{f}_{f},{\eta }_{{CO}_{2}},{f}_{w},{f}_{c}\right)\\ {\Phi }_{3} \left(X\right) =f\left(t, {\eta }_{{CO}_{2}},{DO,f}_{w},{f}_{f},{f}_{e}\right)\end{array}\end{array}\\ {\Phi }_{4} \left(X\right) =f\left(t, {DO,{\eta }_{{CO}_{2}},f}_{w},{f}_{c},{f}_{f}\right)\end{array}\right.$$

### Prediction result analysis

**Fig. 11:**
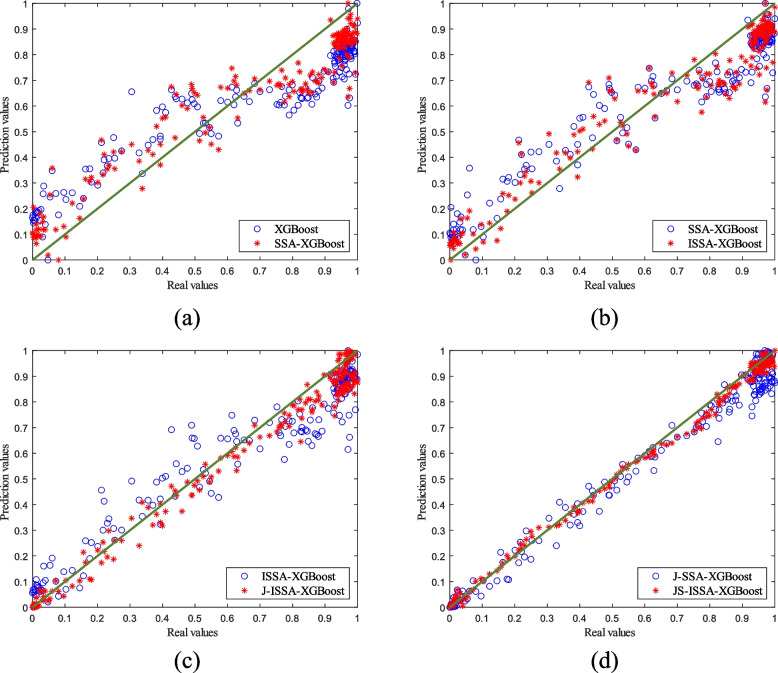
Comparison of cell concentrations predicted by different models for Pichia pastoris

To verify the superiority of the online soft sensor method, we established five models for predicting cell concentration and product concentration in the Pichia pastoris fermentation process: the XGBoost model, SSA-XGBoost model, ISSA-XGBoost model, J-ISSA-XGBoost model, and JS-SSA-XGBoost model. We applied these models to predict the cell concentration, and the results are shown in Fig. 11.

It can be seen from Fig. 11(a) that the cell concentration prediction results of the XGBoost model have a significant degree of dispersion, indicating that the prediction results are very different from the actual values, and the changes in cell concentration cannot be tracked well. In contrast, the improved SSA-XGBoost model using the sparrow optimization algorithm is less discrete because the sparrow algorithm, while limiting the ability of XGBoost to grow freely, improves the ability of XGBoost to generalize partially. As can be seen from Fig. 11(b), the prediction results of the ISSA-XGBoost model are closer to the actual values than the SSA-XGBoost model at some stages after the improvement of the sparrow algorithm. This is because the ISSA optimization algorithm increases the diversity of the model at the later stages of the iteration, which gives the model the ability to jump out of the local optimum. Therefore, the prediction results are improved compared to those before the improvement. Compared with the offline global model in Fig. 11(a) and (b), the prediction ability of the offline global model and the JITL local model can be expressed in Fig. 11(c). In Fig. 11(c), the discreteness of the prediction results of the JITL local model is significantly reduced, which shows that the online modeling scheme is better than the offline modeling. Finally, in Fig. 11(d), it can be seen that the Stacking model built using MLP has improved the prediction accuracy of the model again, which also shows that the MLP model has improved the generalization ability of J-ISSA-XGBoost.

**Fig. 12:**
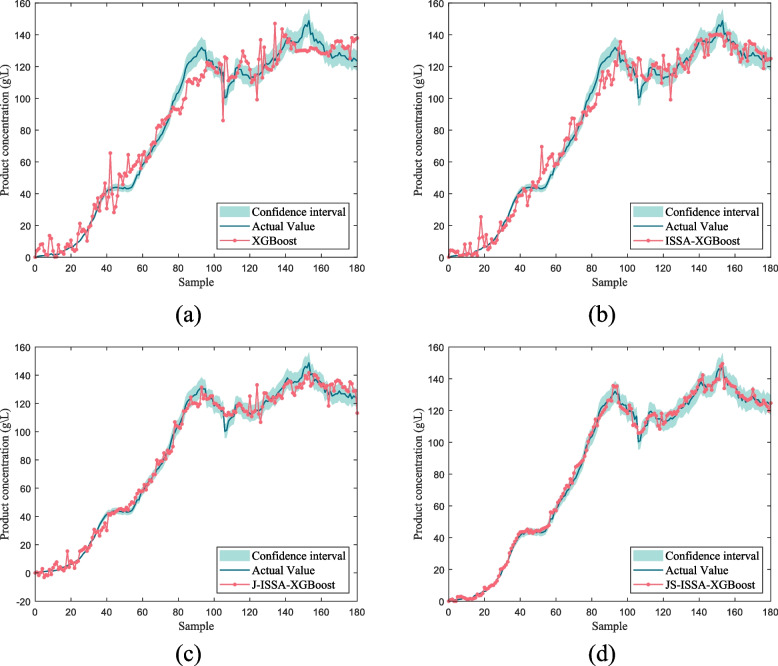
Product concentration prediction curves for different prediction models for Pichia pastoris

To further validate the feasibility of the algorithm, different prediction models were used to predict the product concentration of *Pichia pastoris*, as shown in Fig. 12.

**Fig. 13:**
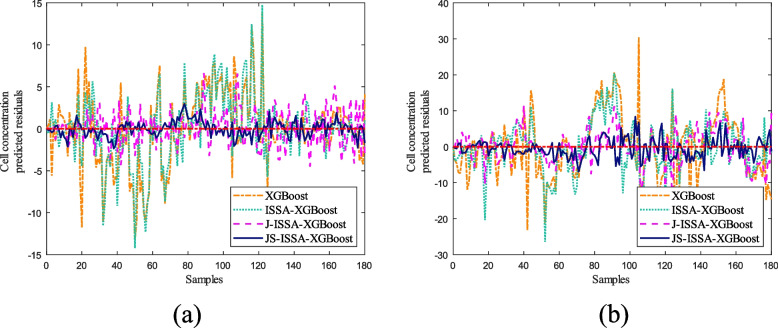
Predicted residual curves of cell concentration versus product concentration under different models

As shown in Fig. 12, the ISSA-XGBoost model optimized in Fig. 12(b) has reduced dispersion in the prediction results compared to the original model in Fig. 12(a). The JITL strategy cited by J-ISSA-XGBoost in Fig. 12(c) predicts significantly lower errors compared to the results predicted by the offline models in Fig. 12(a) and (b). This directly reflects the correctness of the model optimization using the ISSA algorithm and the JITL strategy. Furthermore, as can be seen in Fig. 12(d), the improved JS-ISSA-XGBoost model using the MLP algorithm has higher prediction accuracy compared to the other models throughout the fermentation process of *Pichia pastoris*. In order to better reflect the prediction accuracy of different models, four models were selected for error analysis in this paper, as shown in Fig. 13, and detailed error analysis tables are established, as shown in Table 2.

**Table 2:** Comparison of prediction errors under different models

Model	Cell concentration		Product concentration			
	RMSE	$\boldsymbol{R^2}$	RMSE	$\boldsymbol{R^2}$	MRE	MAE
XGBoost	0.1627	0.7836	9.1977	0.9642	0.4132	30.4069
ISSA-XGBoost	0.1074	0.9058	7.6863	0.9750	0.2898	26.4064
J-ISSA-XGBoost	0.0622	0.9684	4.8525	0.9900	0.2117	17.8598
JS-ISSA-XGBoost	0.0260	0.9945	2.6688	0.9970	0.0889	8.3481

As shown in Table 2, this paper uses root mean square error (RMSE)、coefficient of determination $\left({R}^{2}\right)$, mean relative error (MRE)and maximum absolute error (MAE) as the evaluation index of the model. It can be seen intuitively that the $R^2$ of the improved model has been improved and the RMSE has been reduced significantly. This shows that the JS-ISSA-XGBoost soft sensor model proposed in this paper is superior to other models in terms of prediction accuracy and system robustness.

## Discussion

The biochemical reaction process is nonlinear, time-varying, and coupled. To address this issue, most biochemical reaction processes employ an offline soft sensor modeling approach. Offline models determine parameters by training a historical dataset and fix this portion of parameters. Over time, changes in operating conditions or the environment may render the original parameters unsuitable for the current biochemical reaction process, resulting in the failure of the offline model. The developed JS-ISSA-XGBoost model, on one hand, utilizes the latest data from the current phase and data that significantly contribute to the model from previous phases as the training set, which is then updated in the database. On the other hand, through an online learning approach, the model iterates continuously, constantly updating itself. By implementing the aforementioned improvement approach, the JS-ISSA-XGBoost model resolves the issue of model failure and provides a theoretical foundation for the control of biochemical reactions. In the reaction process, the proposed JS-ISSA-XGBoost model exhibits less variation in prediction accuracy compared to the offline model, making it more suitable for long-term control of biochemical reaction processes.

**Fig. 14:**
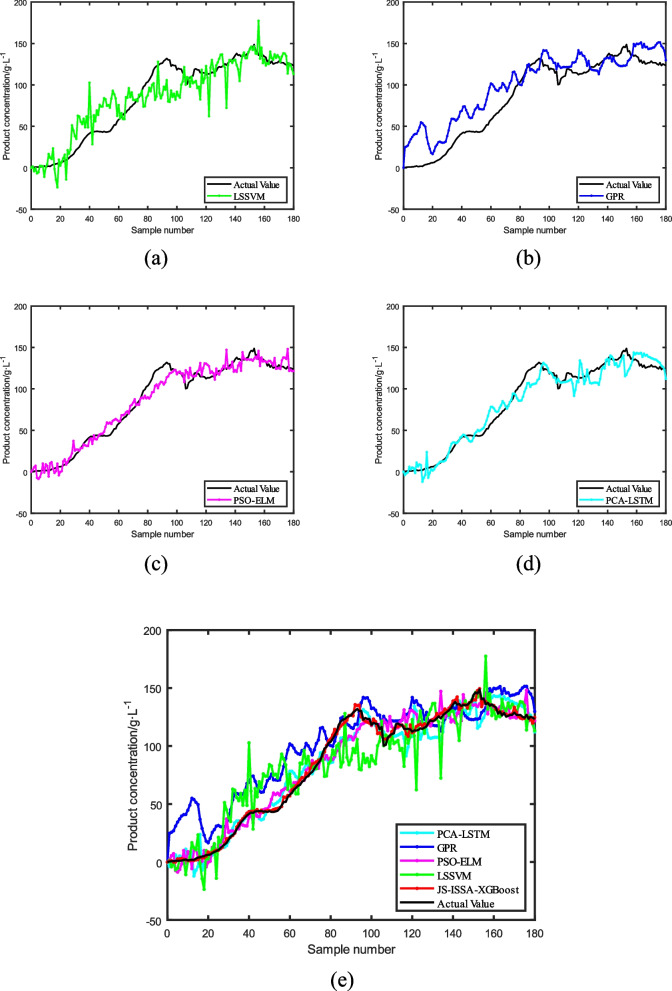
Product concentration curves predicted by different models

**Fig. 15:**
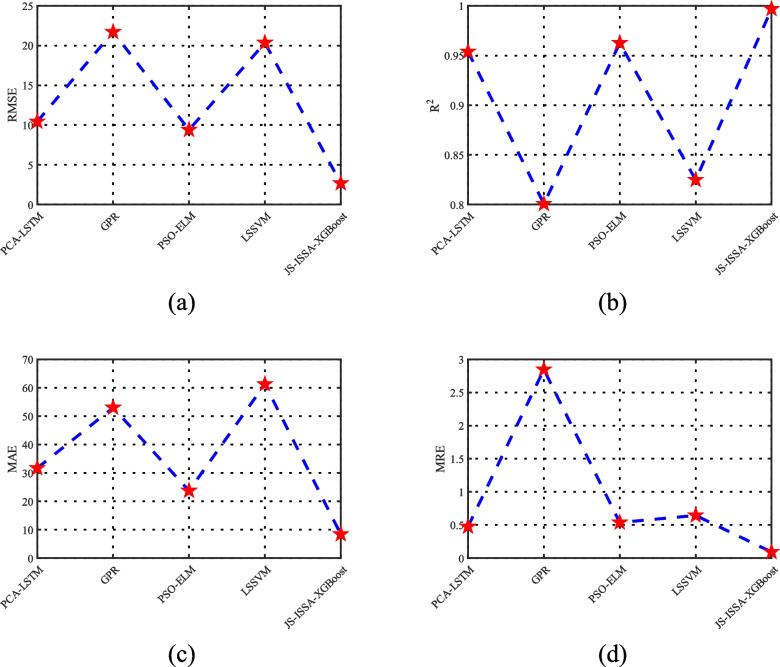
Comparison of performance metrics among different models

Additionally, to further enhance the credibility of the model, this study compares the JS-ISSA-XGBoost model with other algorithmic models, as shown in Fig. 14. As depicted in Fig. 14(a), the LSSVM algorithm, derived from SVM, is suitable for small sample learning. However, there are still large prediction errors in the simulation results, which can have a significant impact on subsequent biochemical process control. As shown in Fig. 14(b), the GPR model exhibits good prediction performance for the reaction process after 80 h of Saccharomyces cerevisiae fermentation, but performs poorly in predicting the reaction process prior to 80 h, indicating its limited stability. As shown in Fig. 14(c) and (d), the PSO-ELM and PCA-LSTM models demonstrate good prediction results with low dispersion, highlighting the feasibility of algorithm-optimized models. Figure 14(e) reveals that the proposed JS-ISSA-XGBoost model outperforms other algorithmic models in terms of superior predictive performance. In this study, various performance metrics were employed to evaluate the performance of the models, as illustrated in Fig. 15. From Fig. 15, it is evident that the JS-ISSA-XGBoost model exhibits smaller values of RMSE, MAE, and MRE compared to other models. Moreover, its $R^2$ value is closer to 1 relative to the other models, indicating that the JS-ISSA-XGBoost model demonstrates superior performance when compared to the other models.

Certainly, the JS-ISSA-XGBoost model also has certain limitations. Compared to offline models, the JS-ISSA-XGBoost model exhibits a certain degree of response lag, which is unavoidable. The JS-ISSA-XGBoost model employs an online learning scheme, which incurs a certain amount of time for searching sample data during real-time learning and iterative model updates. Consequently, the online model exhibits a relative lag compared to the offline model. Furthermore, when applying the JS-ISSA-XGBoost model to different biochemical reaction processes, the number of primary and auxiliary variables needs to be manually determined, which is highly subjective. The variation of auxiliary variables differs significantly across different biochemical reaction processes, and the number of primary and auxiliary variables directly impacts the model's response speed. If the number of auxiliary variables is manually determined to be too high, it increases the complexity of the model, making the solution more challenging and subsequently affecting the response speed of the online model. Conversely, if the number of auxiliary variables is too low, it reduces the model's complexity but results in decreased prediction accuracy. Therefore, the identification of a reasonable number of primary and auxiliary variables through adaptive selection to improve the model's response speed without compromising prediction accuracy poses a significant challenge in optimizing the prediction and control process of biochemical reactions.

In conclusion, in future research, efforts should focus on improving the response speed of online learning models and investigating the adaptive selection of primary and auxiliary variables. It is crucial to strike a balance between the speed and accuracy of online models, thus providing a foundation for further model optimization and systematic prediction control.

## Conclusion

This paper proposes an innovative online soft sensor modeling method based on JS-ISSA-XGBoost, aiming to address the challenges of tracking the dynamic characteristics and parameters of biochemical reaction processes over extended periods. This method constructs multiple sub-databases using offline modeling techniques and adaptively prunes sample data based on similarity labels. In the online modeling stage, the query domain for sampling data is determined using KL divergence, and multiple improved XGBoost soft sensor models are generated using several similarity-driven online learning strategies. Finally, a multilayer perceptron (MLP) is employed to build a stacked ensemble model to enhance the overall algorithm's generalization capability. The proposed method is validated in an actual Pichia pastoris fermentation process, and the experimental results demonstrate high prediction performance with a root mean square error of 0.0260 and a coefficient of determination of 0.9945 for cell concentration, as well as a root mean square error of 2.6688 and a coefficient of determination of 0.9970 for product concentration. These results indicate the model's superior predictive performance.

The JS-ISSA-XGBoost model successfully predicts nonlinear and strongly coupled fermentation processes, demonstrating its high potential in addressing various biochemical reaction problems and can be applied to a wider range of biochemical reaction processes. Furthermore, this online model exhibits advantages in terms of real-time capability, flexibility, and efficiency in biochemical reaction control, which are crucial for optimizing the performance and stability of controllers. This makes it highly suitable for industrial purposes and showcases its immense application prospects.

In the field of process control, in order to enhance the response efficiency of a system, it is imperative to analyze the algorithm's time complexity, memory algorithm complexity, and computational complexity. While the basic XGBoost model has addressed these issues to some extent, we have refrained from providing a detailed explanation in order to prevent the general readers from deviating from the main focus of this paper. However, in the design and application of industrial biochemical reaction control systems, these aforementioned issues merit further in-depth research and exploration.

## Data Availability

The data that support the findings of this study are available from Yangzhong Jiaocheng Biotechnology Research Co., Ltd, but restrictions apply to the availability of these data, which were used under license for the current study, and so are not publicly available. Data are however available from the authors upon reasonable request and with permission of Yangzhong Jiaocheng Biotechnology Research Co., Ltd.

## Abbreviations

JITL: Just-in-time learning
XGBoost: EXtreme Gradient Boosting
FCM: Fuzzy C-means
IFCM: Improved fuzzy c-mean
SSA: Sparrow search algorithm
ISSA: Improved sparrow algorithm
ED: Euclidean distance
CWD: Covariance weighted distance
SSA-XGBoost: Sparrow algorithm to optimize XGBoost models
ISSA-XGBoost: Improved sparrow algorithm for optimizing XGBoost models
J-ISSA-XGBoost: Predictive models based on just-in-time learning
JS-ISSA-XGBoost: Improved just-in-time learning prediction model
MLP: Multilayer perceptron
CV: Cross-validation
K-MI: K-nearest neighbor mutual information estimation
SGD: Stochastic gradient descent
KL: Kullback–leibler divergence
RMSE: Root mean square error
R2: Coefficient of determination
MRE: Mean relative error
MAE: Maximum absolute error
GPR: Gaussian process regression
PSO-ELM: Particle swarm algorithm for optimizing extreme learning machines
LSSVM: Least squares support vector machines
PCA-LSTM: Principal component analysis for optimizing long and short-term memory neural networks
